# α-Glucosidase Inhibition-Guided Network Pharmacology and Molecular Docking Reveal the Antidiabetic Potential of *Cichorium intybus* as a Functional Food

**DOI:** 10.3390/ijms26199497

**Published:** 2025-09-28

**Authors:** Abdul Bari Shah, Aizhamal Baiseitova, Ulpan Amzeyeva, Xiaofei Shang, Janar Jenis

**Affiliations:** 1 College of Pharmacy, Korea University, Sejong 30019, Republic of Korea; abs.uom28@gmail.com; 2 Interdisciplinary Major Program in Innovative Pharmaceutical Sciences, Korea University, Sejong 30019, Republic of Korea; 3 Research Institute for Natural Products & Technology, Almaty 050046, Kazakhstan; ulpan-92.kz@mail.ru; 4 The Research Center for Medicinal Plants, Al-Farabi Kazakh National University, Al-Farabi Ave. 71, Almaty 050040, Kazakhstan; 5 Division of Applied Life Science (BK21 Plus), IALS, Gyeongsang National University, Jinju 52828, Republic of Korea; 6 Kazakh-China Joint Research Center for Natural Veterinary Drug, Almaty 050040, Kazakhstan; shangxiaofei@caas.cn; 7 Lanzhou Institute of Husbandry and Pharmaceutical Sciences, CAAS, Lanzhou 730050, China

**Keywords:** *Cichorium intybus*, α-glucosidase, type 2 diabetes mellitus, network pharmacology, molecular docking

## Abstract

*Cichorium intybus*, commonly known as chicory, is acknowledged as a substitute for coffee and is widely utilized in medicinal applications to treat various ailments. Chicory extract is commonly used in the management of diabetes; however, the specific bioactive components remain unidentified. The present study displayed the antidiabetic potential of chicory using a comprehensive approach integrating in vitro, network pharmacology, and in silico techniques. The methanolic extract demonstrated significant α-glucosidase inhibitory activity in the initial experiment, indicating potential for the management of postprandial hyperglycemia. Based on this, chicory’s major metabolites were identified and examined for their interactions with (type 2 diabetes) T2D targets using network pharmacology. The core genes and pathways involved in the disease were mapped to understand the multitarget mechanisms of the extract. A molecular docking study validated the binding affinity and interactions of leading bioactive compounds with T2D protein targets. The findings indicate that chicory metabolites may serve as promising candidates for the development of natural antidiabetic agents.

## 1. Introduction

*Cichorium intybus* is a well-known medicinal plant, commonly called chicory. It is known as a coffee substitute and is mainly found in Europe and Asia, along with some parts of Africa. Chicory, a hardy plant, can withstand extreme temperatures and produce milky latex when broken [1,2]. This plant comprises four primary cultivars, each with distinct applications. Industrial chicory is utilized for inulin extraction and as a coffee substitute; Brussels chicory is harvested for its etiolated buds; leaf chicory serves as a vegetable, consumed either raw or cooked; and forage chicory, a hybrid of wild chicory, has been utilized since the 1970s to enhance herbage yield in cattle pastures [3]. Chicory has a long history of therapeutic use in various cultures. Its roots have been utilized to treat various symptoms and ailments, including cancer, jaundice, liver disorders, and more [2,4]. More importantly, the ethanolic extract of the whole chicory plant has been extensively studied against diabetes. A study investigated the hypoglycemic and hypolipidemic effects of chicory ethanol extract in the treatment of diabetes. The extract significantly lowered serum glucose levels, triglycerides, cholesterol, and hepatic glucose-6-phosphatase activity in diabetic rats, compared to untreated rats [5]. Another study examines the antidiabetic effects of chicory seed extract on diabetic rats. Results showed that chicory extract prevented weight loss, resisted excessive blood sugar increase, and normalized blood parameters. In early-stage diabetic rats, chicory increased insulin levels. Additionally, its leaf powder diminished blood glucose levels in diabetic Wistar rats, elevated glutathione levels, reinstated anticholinesterase function, and lowered brain lipopolysaccharide and catalase activity [6]. Despite the increasing interest in the therapeutic potential of chicory, thorough mechanistic insights into its bioactive components, particularly through a network pharmacology approach, it remains largely unexamined and under-documented in the scientific literature.

Diabetes mellitus (DM) is a worldwide health issue with growing incidence and mortality rates, especially in low- to middle-income countries. DM affects people of all ages, socioeconomic backgrounds, and demographics [7]. Ancient civilizations acknowledged analogous ailments, and the examination of hyperglycemia facilitated the identification of their causes. DM is a chronic condition resulting from inadequate insulin synthesis or inefficient utilization of the body, leading to hyperglycemia and damage to bodily systems. DM is a complex syndrome characterized by hyperglycemia, classified into type 1 and type 2 diabetes [8,9]. Type 2 diabetes (T2D), a nonautoimmune progressive loss of insulin secretion, often with insulin resistance and metabolic syndrome, accounts for 96% of diabetes cases and poses a substantial threat to human health [10]. The primary pathways to consider in treating diabetes include insulin signaling, insulin resistance, T2D, and the AGE–RAGE signaling pathway in diabetic complications [11,12,13]. Insulin signaling mainly regulates glucose absorption, fat metabolism, and overall energy balance. Insulin resistance diminishes cellular sensitivity to insulin, resulting in the development of T2D and causing persistently elevated blood glucose levels. The AGE–RAGE signaling system, activated in diabetes, primarily result in oxidative stress, inflammation, and vascular damage [11,12,13,14]. These pathways improve our understanding of the fundamental aspects of diabetes and provide significant objectives for effective treatment strategies.

The integration of systems biology and computational approaches has enhanced our understanding of complex diseases like T2D. The exploration of multitarget mechanisms of bioactive compounds through network pharmacology has demonstrated substantial effectiveness. This approach displays a complete “multi-compound, multitarget” paradigm that is more precise for many chronic conditions such as T2D [15,16]. Researchers can identify significant regulatory nodes and signaling pathways influenced by natural compounds by mapping the relationships between phytochemicals and genes or proteins associated with disease [17,18]. This information is necessary to understand how plant extracts work together, to focus on their therapeutic potential, and to find new molecular targets that may not be studied in traditional pharmacological research. On the other hand, molecular docking is a computational method that evaluates the binding interactions between target proteins and bioactive compounds. This study predicts the orientation, binding affinity, and molecular interactions of small compounds with designated proteins, providing understanding about the stability and strength of the compound–target complex [19,20]. Network pharmacology and molecular docking serve as effective alternatives to conventional experimental methods during the initial phases of drug discovery, reducing the likelihood of failure in subsequent stages of development. The growing need for safer, plant-based treatments makes it even more important to use computer-based methods.

The aim of this study is to find out the antidiabetic potential of bioactive compounds in chicory through in vitro, network pharmacology, and molecular docking approaches. The methanolic extract of chicory was evaluated for its α-glucosidase inhibitory activity to show its potential in managing diabetes. Following this, a detailed network pharmacology approach was applied to determine the key bioactive compounds, targeted genes, and the related pathways in diabetes through various online databases. Furthermore, for further confirmation, a molecular docking analysis was performed to examine the interaction between the identified compounds and their target proteins.

## 2. Results

### 2.1. In Vitro α-Glucosidase Inhibitory Activity

**Figure 1:**
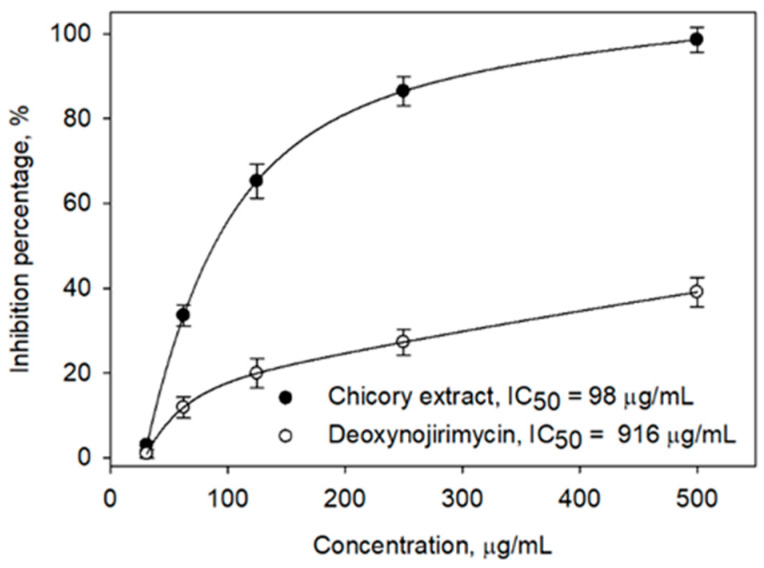
In vitro investigation of chicory extract against α-glucosidase with positive control Deoxynojirimycin.

Numerous studies have demonstrated the antidiabetic potential of chicory extracts, indicating that chicory may have considerable antidiabetic properties. However, the precise mechanisms underlying these effects are not fully understood, as most existing research has concentrated on whole extracts rather than individual bioactive compounds [5,6,21]. This study initially involved the extraction of chicory using methanol, followed by an assessment of its α-glucosidase activity, which exhibited an activity level nearly nine times greater than that of the positive control, Deoxynojirimycin, as illustrated in Figure 1. The in vitro and in vivo analyses previously reported provide compelling evidence that chicory may harbor bioactive compounds with potential applications in diabetes management.

### 2.2. Network Pharmacology In-Depth Analysis

#### 2.2.1. Screening and Selection of Bioactive Compounds in Chicory

**Figure 2:**
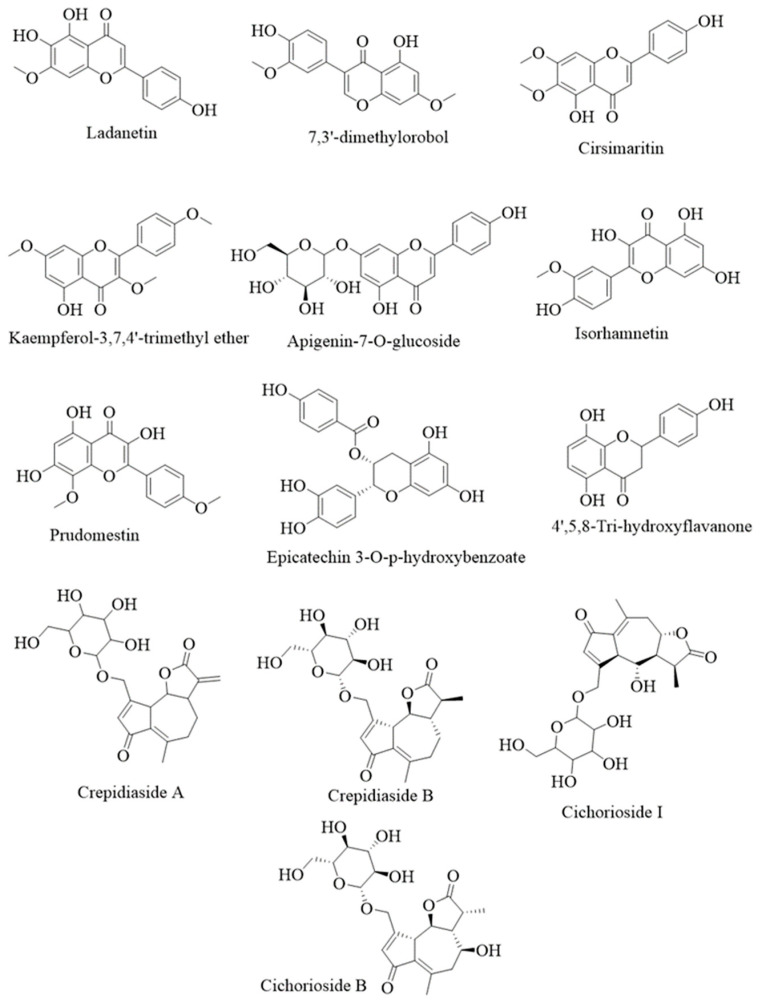
Structures of selected compounds in the initial screening for in-depth network pharmacology analysis.

**Figure 3:**
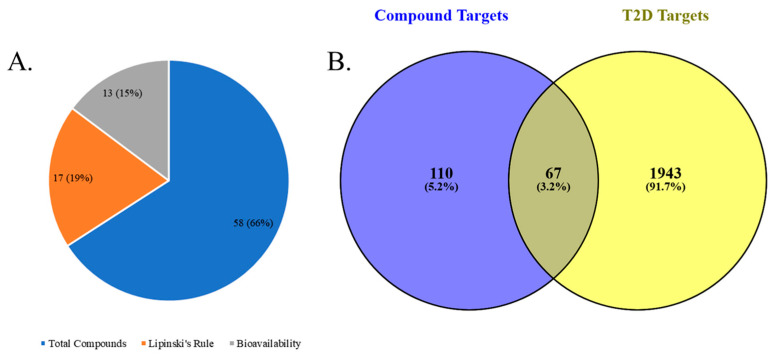
(**A**) Selection of compounds based on Lipinski’s rule and bioavailability. (**B**) Compounds and disease targets with overlapping genes.

The process began with the selection and screening of bioactive compounds from chicory. A total of 59 compounds underwent screening, of which a total of 17 compounds were selected according to Lipinski’s rule. Subsequently, the selected compounds were assessed for their bioavailability score, in which 13 compounds were selected for further analysis. The drug-likeness score, bioavailability, and molecular weight of all compounds are presented in Table 1, their structures are displayed in Figure 2 and the number of selection are presented in Figure 3A. In early drug discovery, drug-likeness and bioavailability ratings are essential metrics, as they indicate whether a compound possesses the necessary physicochemical properties for effective absorption and systemic distribution. Assessing these scores enables researchers to prioritize compounds with higher potential for development into orally active therapeutic drugs [22,23].

#### 2.2.2. Determination of Compound Targets, Disease Targets, and Overlapping Genes

Various online databases were used to identify potential molecular targets of the selected compounds. The study identified 655 distinct target genes across all 13 compounds. Simultaneously, we obtained T2D-related genes from the Human Gene Database, yielding 2010 genes. These genes are associated in various biological processes and signaling pathways associated with the pathophysiology of T2D. An analysis was conducted on the intersections between compound targets and genes related to T2D to evaluate the potential therapeutic relevance of the compounds. The removal of duplicate gene entries from the compound target list resulted in a total of 177 unique genes. The T2D-related gene set was subsequently analyzed for overlapping objectives. We found that 67 genes were common to both the compound targets and the genes associated with T2D (Figure 3B). The presence of overlapping genes suggests potential therapeutic targets, indicating that the selected compounds may positively influence the treatment of T2D by modifying these specific genes.

#### 2.2.3. Protein–Protein Network Analysis

Following the identification of 67 overlapping genes between compounds and T2D, a protein–protein interaction (PPI) analysis was conducted utilizing the STRING database. This analysis seeks to illustrate the functional relationships among these proteins and elucidate their potential roles in T2D treatment.

**Figure 4:**
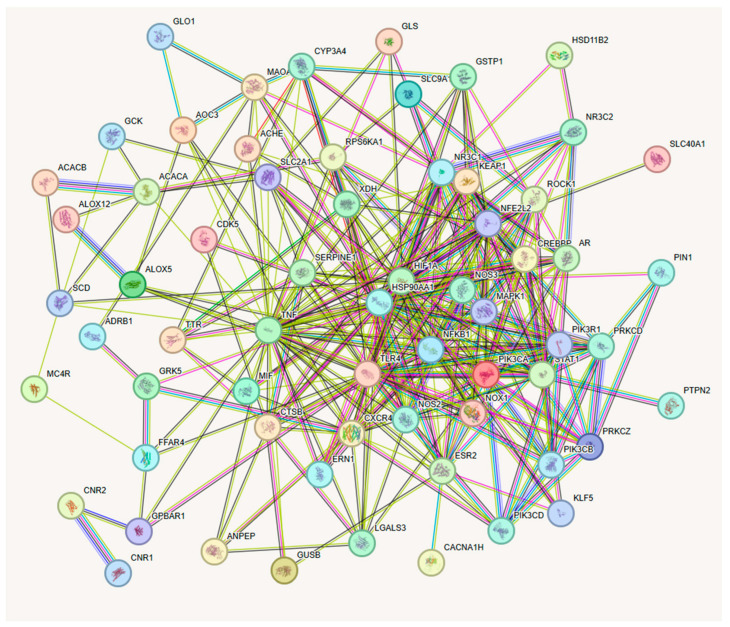
Representation of protein–protein interaction based on overlapping genes through the STRING database. Each node represents a protein, and edges represent predicted or experimentally validated interactions. Different line colors indicate the type of interaction evidence: green (gene neighborhood), red (gene fusion), blue (gene co-occurrence), purple (experimental evidence), yellow (text mining), light blue (curated database annotations), and black (co-expression). Node size is proportional to the degree of connectivity, and edge thickness reflects the confidence score of the interaction.

The interaction network comprised 67 nodes, each representing an overlapping protein. The network consisted of 323 edges, suggesting both direct and indirect connections among the proteins. The average node degree of 9.64 suggests that each protein connects with nearly 10 other proteins in the network. The local clustering coefficient of 0.547 shows a moderate tendency for proteins to form closely linked groups or clusters. The PPI enrichment *p*-value was below 1.0 × 10^−16^, indicating that the observed number of interactions significantly surpasses what would be expected for a random selection of proteins of similar size and degree distribution (Figure 4).

This PPI network illustrates a highly interconnected system of proteins likely involved with common biological processes and signaling pathways associated with T2D. This indicates that these proteins may operate collaboratively or within interrelated pathways, underscoring their potential significance as therapeutic targets for T2D management.

#### 2.2.4. Gene Ontology Enrichment and KEGG Pathway Analyses

To better understand the functional role of the overlapping 67 genes, a Gene Ontology (GO) enrichment analysis was performed. Three main GO categories—biological processes (BP), cellular components (CC), and molecular functions (MF)—were included in this study.

Strict criteria were used in the enrichment study: a false discovery rate (FDR) of less than 0.05, a minimum observed signal greater than 0.01, a minimum enrichment strength greater than 0.01, and a group term similarity score exceeding 0.8. From each GO category, the top 10 most significantly enriched terms were chosen depending on these criteria.

**Figure 5:**
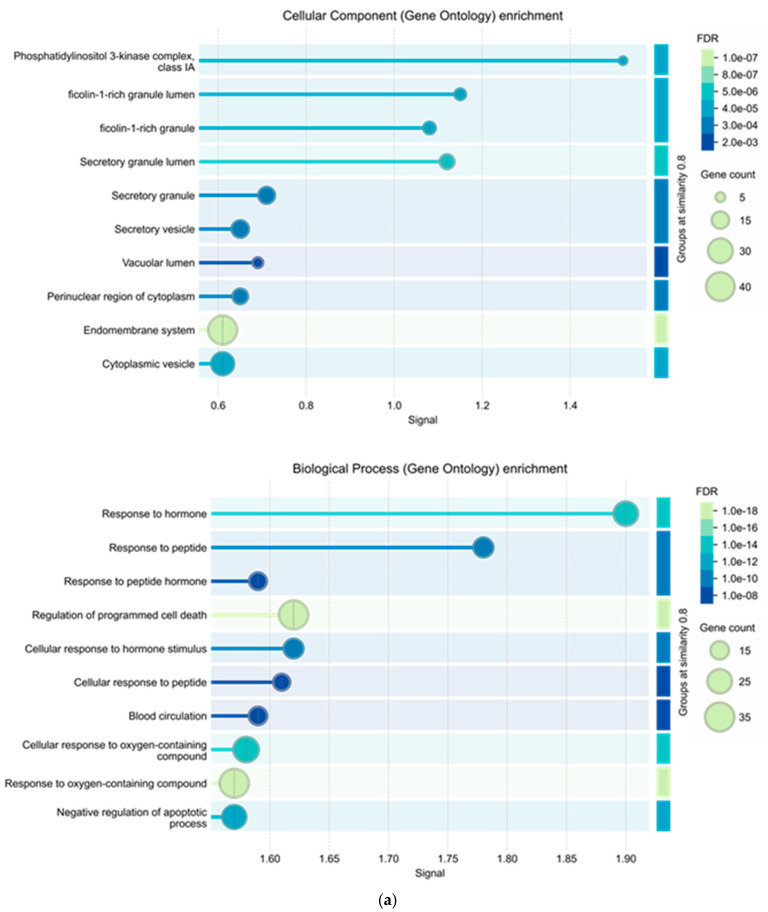

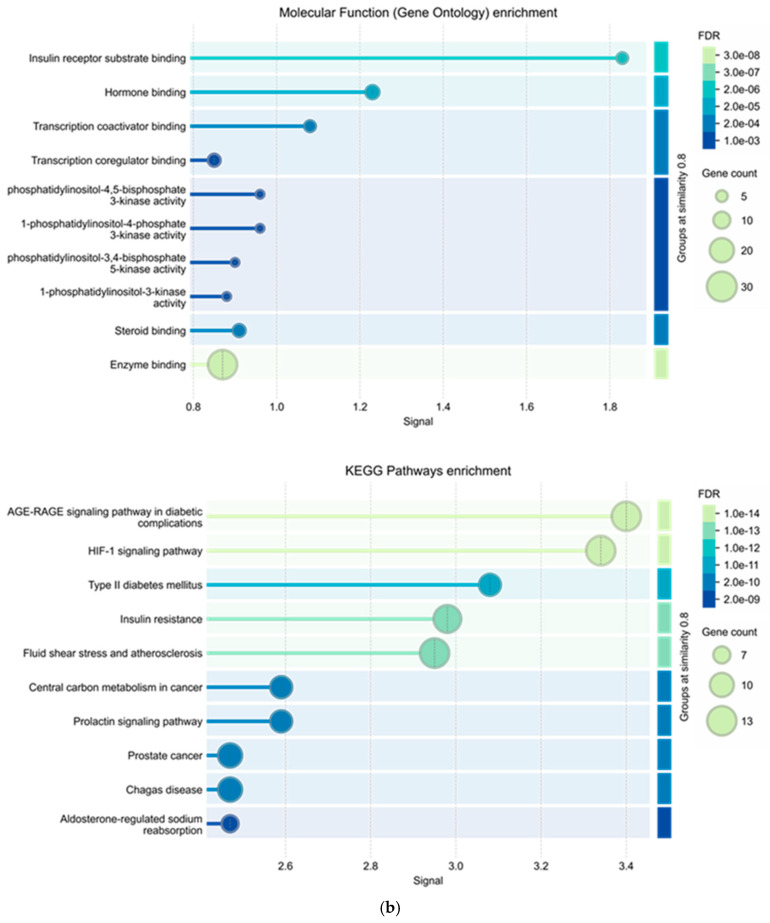
(**a**) Representation of the top 10 components of Gene Ontology enrichment analyses based on the STRING database: Cellular component and Biological process. (Group terms by similarity ≥ 0.8). (**b**) Representation of the top 10 components of Gene Ontology enrichment analyses based on the STRING database: Molecular function and KEGG pathways. (Group terms by similarity ≥ 0.8). Of the selected 4 pathways in diabetes, 3 are present in the top 10.

These enriched GO terms emphasize the main biological functions, subcellular locations, and molecular activities linked to the overlapping genes, emphasizing shared themes like metabolic control, signal transduction, and membrane-associated components, as shown in Figure 5a,b. This implies that by targeting genes involved in major regulatory pathways and cellular activities, the compounds could have therapeutic effects in T2D.

Likewise, KEGG pathway enrichment was performed to examine the signaling pathways associated with the 67 common genes. Figure 5b shows the top 10 enriched pathways. Among these top 10 pathways, 3 are particularly related to T2D. These diabetes-related pathways were selected for further analyses in the following network analyses, offering deeper understanding of the possible mechanisms by which the compounds could have therapeutic effects against T2D.

**Figure 6:**
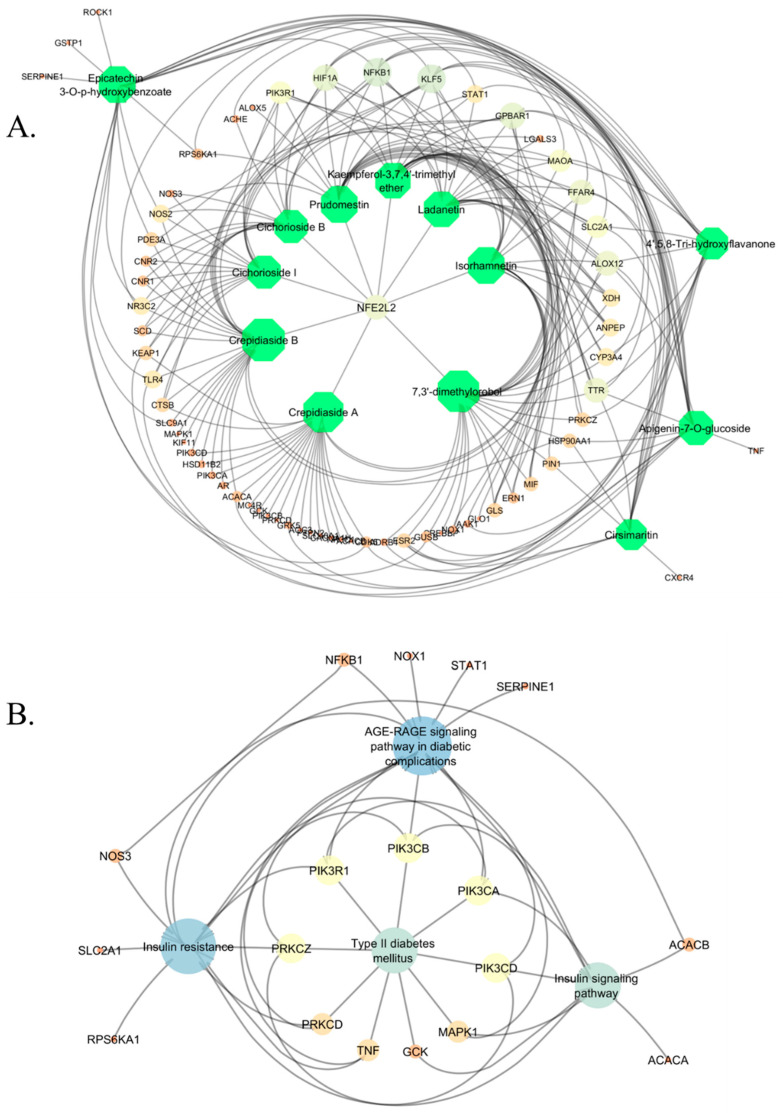
Visualization of (**A**) compound targets, the compounds are represented by hexagons—the more interactions a compound has, the larger its size—and (**B**) pathways targets. All four pathways are presented with bigger circles, the more they interact, the bigger the size of the circle. All colors represent the interactions, with deeper colors indicating stronger interactions and lighter colors indicating weaker interactions.

Additionally, KEGG pathway data relating to the 67 overlapping genes were extracted utilizing the STRING database. This analysis indicated that these genes participate in 149 distinct pathways. Four of the most relevant pathways were selected from these options for further investigation and network construction. The selected pathways encompass the AGE–RAGE signaling pathway in diabetic complications, insulin resistance, type 2 diabetes mellitus, and the insulin signaling pathway. We constructed interaction networks using Cytoscape to visualize the relationships among the compounds, genes, and pathways, as illustrated in Figure 6. Figure 6A depicts the compound–target network. All 13 compounds are presented alongside their corresponding target genes derived from the shared genes. Crepidiaside B targets the most genes, with a total of 24, while both Crepidiaside A and 7,3′-dimethylorobol target 22 genes each. The remaining compounds affect 14 to 19 genes. Figure 6B illustrates the interaction network of pathway target genes. The four selected pathways are interconnected and share several common genes. The AGE–RAGE signaling pathway in diabetic complications is associated with 13 genes, the insulin resistance pathway with 12 genes, and both the type 2 diabetes mellitus and insulin signaling pathways with 9 genes each. Following the elimination of duplicate genes within these pathways, 18 distinct genes were identified. The list comprises *MAPK1*, *SERPINE1*, *NFKB1*, *PIK3CA*, *NOS3*, *PRKCD*, *STAT1*, *NOX1*, *PIK3CD*, *PRKCZ*, *TNF*, *PIK3R1*, *PIK3CB*, *ACACB*, *SLC2A1*, *RPS6KA1*, *GCK*, and *ACACA*. These genes are essential components of the network and may function as viable targets for T2D treatment, underscoring the potential multitarget mechanisms of the chosen compounds.

**Figure 7:**
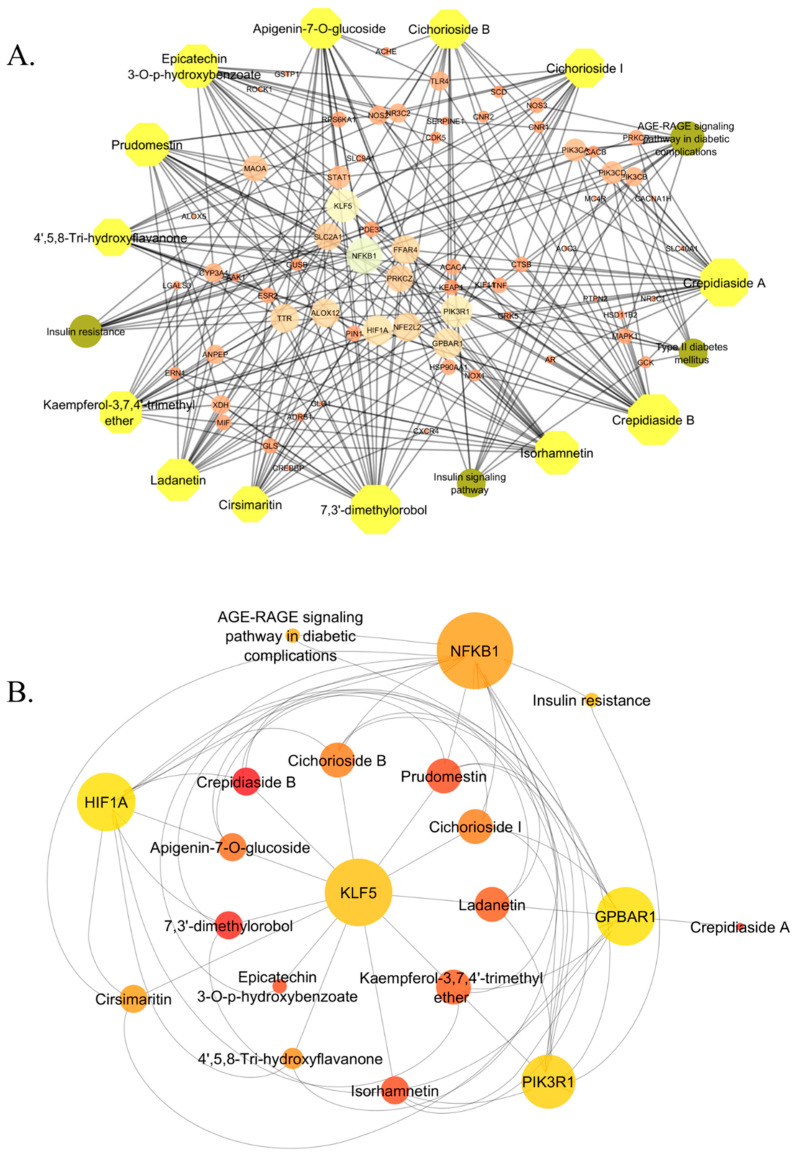
(**A**) Merged network of compounds and pathway targets. Thirteen selected compounds were associated with 240 possible targets and four distinct pathways significant to diabetes. (**B**) Representation of the top 20 ranked compounds and genes inside the merged network of compounds and pathway targets. All colors represent the interactions, with deeper colors indicating stronger interactions and lighter colors indicating weaker interactions.

To facilitate a more comprehensive analysis, we developed a merged network containing both compounds and pathways targets, as illustrated in Figure 7A. The integrated network indicates extensive interrelation among genes, pathways, and compounds. The compounds in this network are represented as hexagons in a greenish color, pathways are illustrated as circles in a purple shade, and the remaining circles denote genes. The larger the circle, the greater the interaction. It is evident that certain gene circles are bigger than others due to their stronger interactions and involvement within this network. To identify the highest-ranked interactions, we apply the cytoHubba plugin and determined the top 20 interactions, as illustrated in Figure 7B. In combination with 13 compounds, the five genes *NFKB1*, *PIK3R1*, *KLF5*, *GPBAR1*, and *HIF1A*, along with the two pathways AGE–RAGE signaling pathway in diabetes complications and insulin resistance, established the top 20 ranked relationships. The genes *NFKB1*, *PIK3R1*, *KLF5*, *GPBAR1*, and *HIF1A* play a critical role in the onset and progression of T2D by participating in essential metabolic and inflammatory pathways. *NFKB1* is notably linked to insulin resistance and T2D, highlighting its potential as a therapeutic target. This process enhances the expression of pro-inflammatory cytokines in reaction to hyperglycemia and lipid accumulation, thus mediating inflammation primarily linked to insulin resistance and beta-cell dysfunction [24,25]. *PIK3R1* is important for insulin signaling, because it codes for a regulatory subunit of the PI3K enzyme. Its disfunction disturbs the PI3K/AKT pathway, which makes it harder for cells to take up glucose and respond to insulin [26,27]. The remaining three genes (*KLF5*, *GPBAR1*, and *HIF1A*) are also related with diabetes, which can be seen in numerous studies [28,29,30,31,32,33]. These genes show how inflammation, lipid regulation, and poor signaling all work together in the pathophysiology of T2D. After this description, the two genes linked to T2D, *NFKB1*, and *PIK3R1*, were chosen for molecular docking studies to see how they interact with certain drugs. Twelve of the thirteen compounds targeted *NFKB1*, and seven targeted *PIK3R1*. In the next section, molecular docking studies will confirm their interactions.

**Figure 8:**
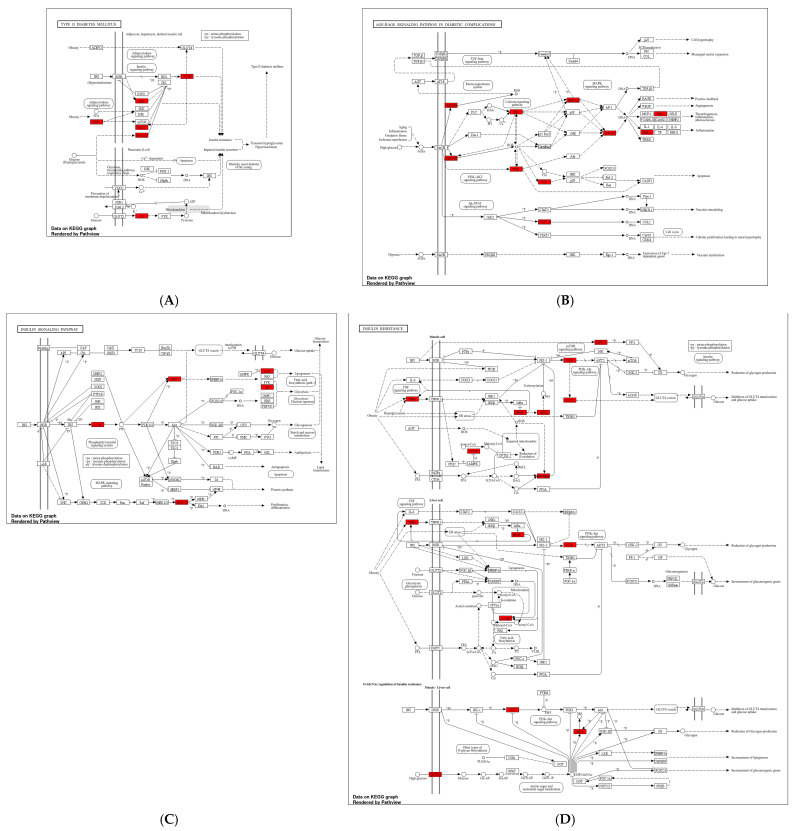
The selected pathways are targeted by selected compounds. The target genes are highlighted in red. The FDR cutoff value was 0.05. (**A**) Type 2 diabetes mellitus; (**B**) AGE–RAGE signaling pathway in diabetic complications; (**C**) insulin signaling pathway; (**D**) insulin resistance pathway.

Furthermore, we analyzed the selected KEGG pathways using the ShinyGO online database by inputting the overlapping genes. The targeted genes in our selected pathways (AGE–RAGE signaling pathway in diabetic complications, insulin resistance, type 2 diabetes mellitus, and the insulin signaling pathway) are highlighted in red, as shown in Figure 8.

This study carefully identified and structured bioactive compounds from chicory that exhibit potential antidiabetic effects using an integrated network pharmacology approach. Of the 59 compounds initially screened, 13 fulfilled the criteria for drug-likeness and bioavailability, aligning with previous research highlighting the significance of physicochemical properties in oral drug development [16,34]. The identification of 67 overlapping genes between chicory-derived targets and T2D-associated genes highlights the multitarget potential of chicory constituents. The PPI network demonstrated highly interconnected protein clusters, indicating that these compounds may influence multiple pathways concurrently instead of targeting a single pathway. The multitarget approach is increasingly acknowledged as beneficial in the management of complex diseases such as T2D, characterized by concurrent dysregulation of insulin signaling, inflammation, and oxidative stress [35,36,37].

The GO and KEGG enrichment analyses identified significant pathways, such as AGE–RAGE signaling, insulin resistance, type 2 diabetes mellitus, and insulin signaling, with three of these pathways ranking in the top ten, thereby underscoring their importance in T2D pathology. The findings are consistent with prior research indicating the modulation of these pathways by bioactives derived from plants [37,38,39]. Notably, *NFKB1* and *PIK3R1* emerged as central hub genes, both of which have been implicated in glucose homeostasis and inflammatory processes associated with T2D [25,27]. The following molecular docking validation demonstrated robust interactions between these pivotal genes and several chicory compounds, hence reinforcing their therapeutic promise. Collectively, our findings indicate that the bioactives of chicory may provide synergistic effects by concurrently modulating many signaling pathways, a strategy that could potentially address the shortcomings of traditional monotherapies.

Based on this comprehensive description from network pharmacology, it is evident that the bioactive compounds in chicory possess the potential to cure T2D; however, further in vivo research is required to investigate these target genes associated with individual compounds to validate their efficacy.

### 2.3. Molecular Docking Studies

**Figure 9:**
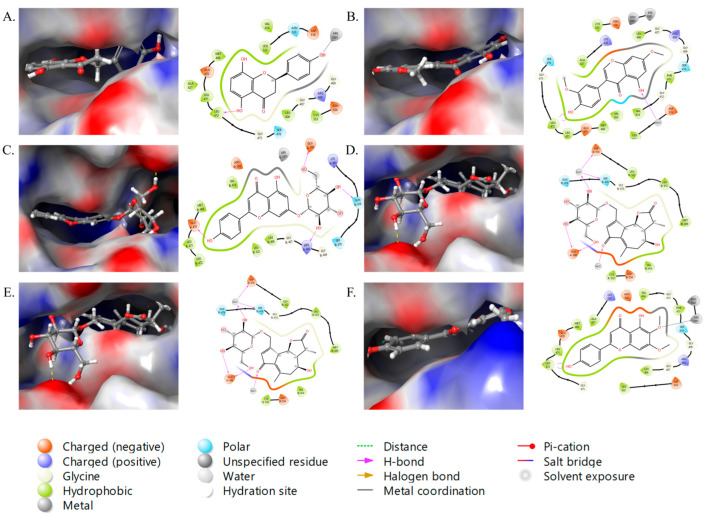
Docking studies with 3D and 2D representation of *NFKB1* against (**A**) 4′,5,8-tri-hydroxyflavanone; (**B**) 7,3′-dimethylorobol; (**C**) apigenin-7-O-glucoside; (**D**) cichorioside B; (**E**) cichorioside I; (**F**) cirsimaritin.

**Figure 10:**
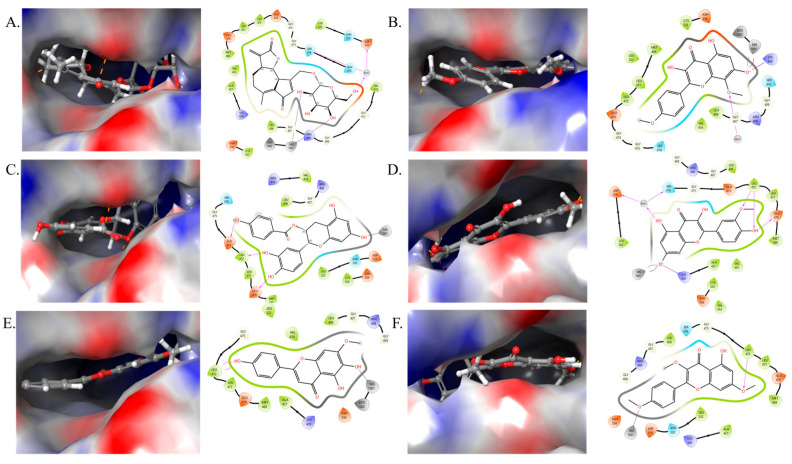
Docking studies with 3D and 2D representation of *NFKB1* against (**A**) crepidiaside A; (**B**) prudomestin; (**C**) epicatechin 3-O-p-hydroxybenzoate; (**D**) isorhamnetin; (**E**) ladanetin; (**F**) kaempferol-3,7,4′-trimethyl ether.

We have analyzed the two significant targets (*NFKB1* and *PIK3R1*) identified through our network pharmacology investigations. *NFKB1* is a gene that encodes the p105 protein, which is a precursor to the p50 subunit of the NF-κB transcription factor. It mainly controls inflammation, cell development, and immunity. *NFKB1* participates in many cellular processes, including stress response, natural and adaptive immunity, and apoptosis. *NFKB1* is linked to the onset and progression of T2D because of its involvement in inflammation and insulin resistance, especially its part in the inflammatory response mediation, which are central to T2D pathogenesis [24,40]. In the current study, out of 13 compounds, 12 targeted the *NFKB1*,highlight it as a major therapeutic target of chicory. To confirm the binding of these compounds with *NFKB1*, we obtained the crystal structure of *NFKB1* from the Protein Data Bank and studied the molecular docking analysis of all 12 compounds by using the Maestro v12.4 (Schrodinger, New York, NY, USA). The 2D and 3D representations of all 12 compounds with their binding analysis are shown in Figure 9 and Figure 10. The different interactions among the compounds and protein that include hydrogen bonding, hydrophobic interaction, pi–pi stacking, etc., are shown in the respective figures.

Among the tested compounds, isorhamnetin and prudomestin displayed the best glide score with −11.3776 and −9.66427 kcal/mol, respectively. Isorhamnetin is an O-methylated flavonol from the class of flavonoids. It is found in many other plants, including *Hippophae rhamnoides* L., *Ginkgo biloba* L., *Elaeagnus rhamnoides* (L.) A. Nelson, and many other plants [41,42]. Isorhamnetin has been investigated for its potential advantages in several domains, including cardiovascular and cerebrovascular protection, anti-tumor effects, anti-inflammatory capabilities, antioxidant activity, and obesity prevention, via processes associated with signaling pathways and cytokine expression [42]. Prudomestin, a hydroxyflavan, has been reported previously in *Prunus domestica* [43] and *Zanthoxylum armatum* with antioxidant activities [44], and can also be synthesized in laboratory conditions [45]. The apigenin-7-O-glucoside, cirsimaritin, crepidiaside A, epicatechin 3-O-p-hydroxybenzoate, and ladanetin also displayed a good binding affinity with −8.05496, −8.49519, −8.14023, −8.15487, and −8.76043 kcal/mol, respectively. While the remaining compounds—4′,5,8-tri-hydroxyflavanone, 7,3′-dimethylorobol, cichorioside B, cichorioside I, and kaempferol-3,7,4′-trimethyl ether—exhibited a rather strong binding affinity with glide scores of −7.53832, −7.35084, −6.10589, −7.7162, and −6.48179 kcal/mol, respectively (Table 2). Overall, these compounds demonstrate a significant binding affinity with *NFKB1*, indicating their potential as therapeutic agents for T2D. However, further experimental validation is required to confirm these findings.

**Figure 11:**
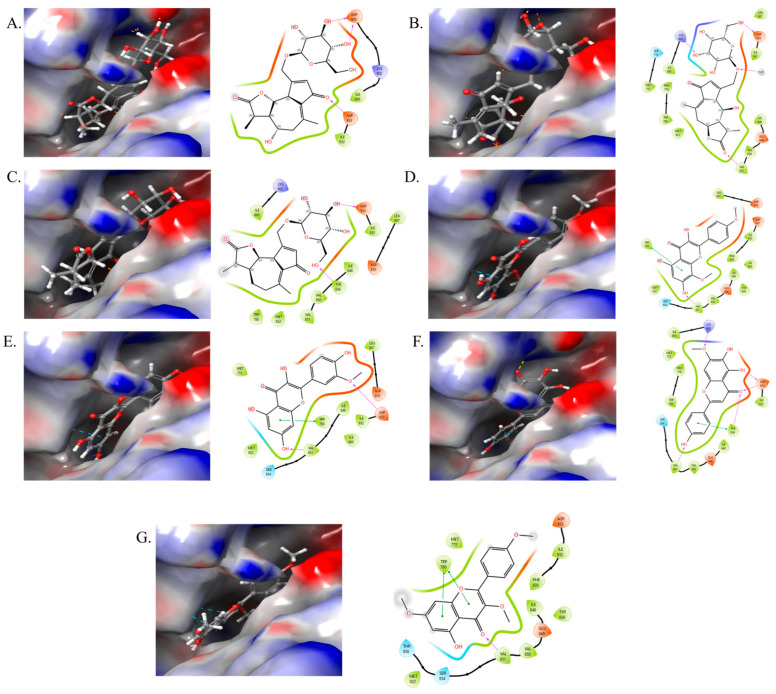
Docking studies with 3D and 2D representation of *PIK3R1* against (**A**) cichorioside B; (**B**) cichorioside I; (**C**) crepidiaside B; (**D**) prudomestin; (**E**) isorhamnetin; (**F**) ladanetin; (**G**) kaempferol-3,7,4′-trimethyl ether.

In a similar way, the *PIK3R1* was also subjected to molecular docking studies. *PIK3R1*, commonly referred to as p85α, serves as a regulatory subunit of phosphoinositide 3-kinases (PI3Ks), facilitating the phosphorylation of phosphatidylinositol into secondary signaling molecules. The conversion of phosphatidylinositol 4,5-bisphosphate (PIP2) to phosphatidylinositol 3,4,5-triphosphate (PIP3) facilitates the recruitment of protein kinase AKT to the cell membrane, thereby playing a crucial role in metabolic functions. *PIK3R1* serves to stabilize and inhibit the catalytic activity of p110 while also engaging with insulin receptor substrate proteins and growth factor receptors. It plays an important role in the regulation of insulin signaling, thereby having a pivotal role in diabetes [27]. Out of 13 compounds, 7 displayed a good binding affinity (Figure 11), as all 7 of these compounds targeted the *PIK3R1* in the network pharmacology analysis. The highest binding affinity with good glide score was displayed by ladanetin, cichorioside I, and isorhamnetin with −9.36, −8.62, and −8.11 kcal/mol, respectively (Table 3).

Ladanetin is an active flavonoid and is reported from *Sesamum indicum* [46], *Halophila johnsonii* [47], and chicory. Cichorioside I is a terpene glycoside, although there is very limited literature available on this compound. The remaining compounds cichorioside B, crepidiaside B, prudomestin, and kaempferol-3,7,4′-trimethyl ether, displayed a binding affinity with a glide score of −7.16, −7.00, −7.57, and −7.03 11 kcal/mol, respectively. All seven compounds exhibit significant binding affinities, indicating their potential as effective therapeutic agents for T2D, necessitating additional research.

The identification of *NFKB1* as a major hub gene based on our network pharmacology study presents its central role in T2D pathophysiology. *NFKB1* encodes the precursor protein p105, which is then processed to form the p50 subunit of NF-κB, a master transcription factor regulating inflammation, apoptosis, and immune responses. Chronic low-grade inflammation is a hallmark of T2D, and *NFKB1* is a key mediator linking hyperglycemia to inflammatory cytokine expression and insulin resistance [27,48,49,50]. Our results demonstrated that 12 of the 13 selected chicory compounds target *NFKB1*, suggesting that chicory’s bioactives may exert anti-inflammatory and insulin-sensitizing effects through NF-κB modulation. Molecular docking studies confirmed strong binding affinities, with isorhamnetin and prudomestin showing the highest glide scores (−11.37 and −9.66 kcal/mol, respectively). Isorhamnetin has been previously reported for its anti-inflammatory and metabolic regulatory effects, supporting our findings. This strong binding affinity suggests that isorhamnetin and other chicory compounds could effectively inhibit NF-κB activation, potentially reducing cytokine-driven inflammation and improving insulin sensitivity, an important therapeutic strategy in T2D management.

In a similar way, *PIK3R1* emerged as another key target, reflecting its pivotal role and involvement in insulin signaling and glucose homeostasis. It encodes the p85α regulatory subunit of PI3K, which stabilizes and activates the catalytic subunit, leading to AKT phosphorylation and downstream effects on glucose uptake. Dysregulation of *PIK3R1* has been associated with impaired insulin signaling and glucose intolerance [27,51,52]. In our study, seven compounds displayed significant binding affinities with *PIK3R1*, with ladanetin (−9.36 kcal/mol), cichorioside I (−8.62 kcal/mol), and isorhamnetin (−8.11 kcal/mol) demonstrating the strongest interactions. The concurrent targeting of *NFKB1* and *PIK3R1* by various drugs is notably promising, indicating simultaneous regulation of inflammatory and insulin signaling pathways. This multitarget strategy may yield synergistic advantages by diminishing inflammation and reinstating insulin sensitivity, presenting a possible therapeutic edge over single-target antidiabetic drugs. These findings necessitate additional in vitro and in vivo confirmation, along with mechanistic investigations to elucidate how these interactions influence functional consequences in glucose management and metabolic health.

The docking scores shown in the study are biologically significant, presenting preliminary evidence that chicory-derived compounds may interact stably with key proteins involved in the pathogenesis of T2D. Lower (more negative) docking scores typically indicate stronger binding affinity and an increased probability of biological activity. The values obtained in this study, specifically for isorhamnetin (−11.37 kcal/mol) with *NFKB1* and ladanetin (−9.36 kcal/mol) with *PIK3R1*, align with the range documented for recognized bioactive phytochemicals exhibiting therapeutic effects. Strong binding interactions, including hydrogen bonding, π–π stacking, and hydrophobic interactions, were commonly observed, indicating that these compounds may efficiently occupy the active or regulatory sites of these proteins. Stable binding may inhibit NF-κB activation and enhance PI3K/AKT signaling, resulting in decreased pro-inflammatory cytokine expression and improved glucose uptake. The findings display the hypothesis that chicory bioactives work via a multitarget mechanism, which is especially relevant for the management of a multifactorial disease such as T2D.

Research outputs from molecular docking demonstrated that the selected and analyzed chicory-derived compounds exhibit notable interactions with their specific target proteins. Subject to further experimental validation, these findings suggest that the examined compounds may serve as effective therapeutic agents for the control of T2D.

## 3. Materials and Methods

### 3.1. Plant Material

Roots of *Cichorium intybus* (chicory) were obtained from Jiangsu Yabang Chinese Medicine Co., Ltd. (Changzhou, Jiangsu, China). A voucher specimen (KAZNU-202403) has been preserved at the Research Center for Medicinal Plants, Al-Farabi Kazakh National University, Almaty, Kazakhstan.

### 3.2. α-Glucosidase Inhibitory Activity

The inhibitory effect on α-glucosidase was according to previous research with little modifications [53]. The methanolic extract of chicory was first dissolved in dimethyl sulfoxide (DMSO) and subsequently diluted to the desired concentration. α-Glucosidase, which was obtained from *Saccharomyces cerevisiae* (Sigma Aldrich, St. Louis, MO, USA), and a substrate known as p-nitrophenyl-α-D-glucopyranoside, were both employed along with a 50 mM potassium phosphate buffer with a pH of 6.8 for the assay. Within a 96-well plate, the reaction mixture was prepared using 130 µL of buffer, 10 µL of either sample or DMSO, 40 µL of substrate with a concentration of 200 µL, and 20 µL of enzyme with a concentration of 0.01 U/mL. (All chemicals were purchased from Sigma Aldrich). Following enzyme addition, the absorbance was immediately measured at 405 nm at 37 °C for 30 min. Enzyme inhibition was quantified using IC_50_ values. The following equation was employed to obtain the percentage inhibition ratio:

Activity (%) = 100 [1/(1 + ([I]/IC_50_))] (1)

### 3.3. Network Pharmacology

#### 3.3.1. Screening of Compounds of Chicory

We previously performed a detailed LCMS analysis of chicory extract and successfully identified 59 distinct compounds [54]. SMILES representations of the compounds were generated with ChemDraw 19.0 and subsequently entered into the MolSoft database (https://www.molsoft.com/mprop/, accessed on 24 March 2025) for the evaluation of drug-likeness scores, number of hydrogen bond donors and acceptors, MolLogP, MolLogS, MolPSA, and additional parameters. Compounds with scores ≥ 0.18 and no more than three violations were selected. Additionally, the online database http://www.swissadme.ch/ (accessed on 24 March 2025) was utilized to determine the bioavailability of compounds. The bioavailability threshold was chosen to be ≥30% (0.3) [55,56]. The details information are available in Supplementary Materials.

#### 3.3.2. Identification of Selected Compound Targets and Disease Target Genes

The SuperPred online tool (https://prediction.charite.de/index.php, accessed on 26 March 2025 ) was utilized to predict the probable targets of the chosen compounds, and UniProt IDs were obtained for those with a probability score of ≥65%, thereby providing a high level of confidence. Thereafter, the STRING database (https://string-db.org/ accessed on 26 March 2025) was used to examine the functional relationships and interaction networks of the projected targets, providing significant insights into their potential biological roles and pathways. For the disease targets, https://www.genecards.org/ (accessed on 28 March 2025) was used by inserting the keyword “T2D”. A filter threshold of ≥30 was applied to disease target genes. The tool https://bioinfogp.cnb.csic.es/tools/venny/ (accessed on 28 March 2025) was used to determine the common genes between the compounds and T2D. The Venn diagram was used to display the common genes for all compounds individually and also for the total compounds against disease target genes. The common or overlapping genes were used in further analysis [57,58,59].

#### 3.3.3. Determination of Protein–Protein Interaction and Gene Ontology

The STRING database (https://string-db.org/ accessed on 28 March 2025) was used for the analysis of protein–protein interaction (PPI) through the input of the common genes. Homo sapiens was selected as the target species, with a confidence level of 0.900 established. The gene ontology was established through the database through performing the top 10 enrichment studies. The KEGG pathways were examined, and the most significant pathways related to T2D were found. Additionally, the ten most prominent pathways were selected for inclusion in the enrichment analysis [55,58].

#### 3.3.4. Visualization by Cytoscape

The chosen pathways and compound targets were then visualized using Cytoscape 3.7. A distinct network was established, incorporating pathway targets, compound targets, and their integrated network, where both compounds and diseases are interrelated. The cytoHubba plugin was utilized to identify the highest-ranked interactions inside the merged network [60,61].

To emphasize the relevant targeted genes inside KEGG pathways, ShinyGO 0.81 (https://bioinformatics.sdstate.edu/go/, (accessed on 29 March 2025) was utilized. The genes marked in red and the most pertinent pathways chosen for network visualization were selected [62].

### 3.4. Molecular Docking

The crystal structures utilized for molecular docking studies include *NFKB1* (PDB-ID: 4DN5) and *PIK3R1* (PDB-ID: 5XGJ), sourced from the Protein Data Bank (https://www.rcsb.org/). To optimize the protein structure, the “Orthogonal Partial Least Squares 4” (OPLS4) potential energy function and the protein preparation tool in Maestro v12.4 (Schrodinger, New York, NY, USA) were used until the average RMSD of non-hydrogen atoms reached 0.3 Å. For ligand structure creation, ChemDraw 19.0 PerkinElmer Informatics was used and were converted to 3D structures using the LigPrep tool. The Receptor Grid Generation function in Maestro v12.4 (NM share, version 6.0.134; Schrödinger) was used to define the docking grid for the receptor’s active site using coordinates from the PDB file, thereby producing the receptor grid. The grids were formed at the sites of co-crystallized ligands. The site was established using the following coordinates: X29.88_Y46.11_Z40.0 for 5XGJ, and X6.37_Y51.74_Z-20.09 for 4DN5, which were used to construct the core of the receptor grid box. The grid box dimensions were modified to align with those of the co-crystallized ligand. Glide in XP mode was then used for docking and calculations.

## 4. Conclusions

Chicory is an important medicinal plant characterized by a diverse phytochemical composition and various pharmacological properties, such as antidiabetic, anti-inflammatory, and antioxidant effects. This research presents a thorough examination of chicory compounds in relation to type 2 diabetes (T2D) through in vitro assays, network pharmacology, and molecular docking approaches. The preliminary analysis indicated that the methanolic extract of chicory demonstrated notable inhibitory activity against α-glucosidase, an essential enzyme in T2D. Utilizing network pharmacology, we examined 59 compounds and identified 13 as the most promising candidates for drug development, based on their drug-likeness and bioavailability scores. The potential targets and related T2D genes were systematically investigated using PPI network construction, gene ontology, and KEGG pathway analyses. Four primary pathways were identified: AGE–RAGE signaling, insulin resistance, T2D, and insulin signaling. Three of these pathways ranked among the top ten enriched pathways, thereby further validating the antidiabetic potential of chicory bioactives. NKB1 and *PIK3R1* were prioritized for molecular docking studies due to their significant binding interactions with selected compounds, thereby reinforcing the findings from network pharmacology. The results underscore the therapeutic potential of chicory bioactive compounds in managing T2D and highlight the necessity for additional pharmacological and clinical studies to validate their efficacy and safety.

## Figures and Tables

**Table 1:** Selected compounds in chicory for network pharmacology analysis.

S. No.	Compounds Name	Molecular Weight	Drug Likeness Score	Bioavailability
1	Ladanetin	300.06	0.49	0.55
2	7,3′-dimethylorobol	314.08	0.26	0.55
3	Cirsimaritin	314.08	0.47	0.55
4	Kaempferol-3,7,4′-trimethyl ether	328.09	0.21	0.55
5	Apigenin-7-O-glucoside	432.11	0.59	0.55
6	Isorhamnetin	316.06	0.39	0.55
7	Prudomestin	330.07	0.25	0.55
8	Epicatechin 3-O-p-hydroxybenzoate	410.10	0.90	0.55
9	4′,5,8-Tri-hydroxyflavanone	272.07	0.30	0.55
10	Crepidiaside A	422.16	0.54	0.55
11	Crepidiaside B	424.17	0.79	0.55
12	Cichorioside I	440.17	0.72	0.55
13	Cichorioside B	440.17	0.76	0.55

**Table 2:** Docking studies of compounds against *NFKB1*.

S. No.	Compounds	*NFKB1* (Glide Score)
1	4′,5,8-tri-hydroxyflavanone	−7.53832
2	7,3′-dimethylorobol	−7.35084
3	Apigenin-7-O-glucoside	−8.05496
4	Cichorioside B	−6.10589
5	Cichorioside I	−7.7162
6	Cirsimaritin	−8.49519
7	Crepidiaside A	−8.14023
8	Prudomestin	−9.66427
9	Epicatechin 3-O-p-hydroxybenzoate	−8.15487
10	Isorhamnetin	−11.3776
11	Ladanetin	−8.76043
12	Kaempferol-3,7,4′-trimethyl ether	−6.48179

**Table 3:** Docking studies of compounds against *PIK3R1*.

S. No.	Compounds	*PIK3R1* (Glide Score)
1	Cichorioside B	−7.16
2	Cichorioside I	−8.62
3	Crepidiaside B	−7.00
4	Prudomestin	−7.57
5	Isorhamnetin	−8.11
6	Ladanetin	−9.36
7	Kaempferol-3,7,4′-trimethyl ether	−7.03

## Data Availability

All data supporting the findings of this study are provided in the main manuscript and Supplementary Materials. Any additional datasets or information can be obtained from the corresponding author upon reasonable request.

## Supplementary Materials

The following supporting information can be downloaded at: https://www.mdpi.com/article/10.3390/ijms26199497/s1.
